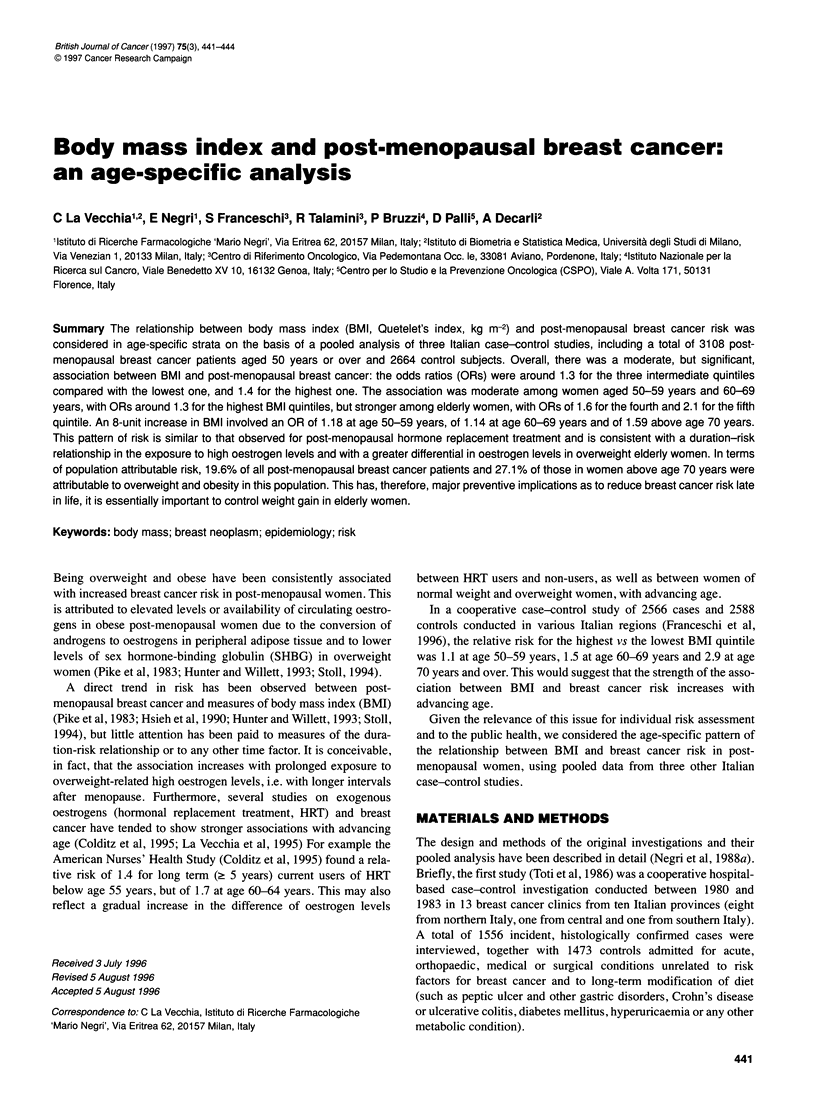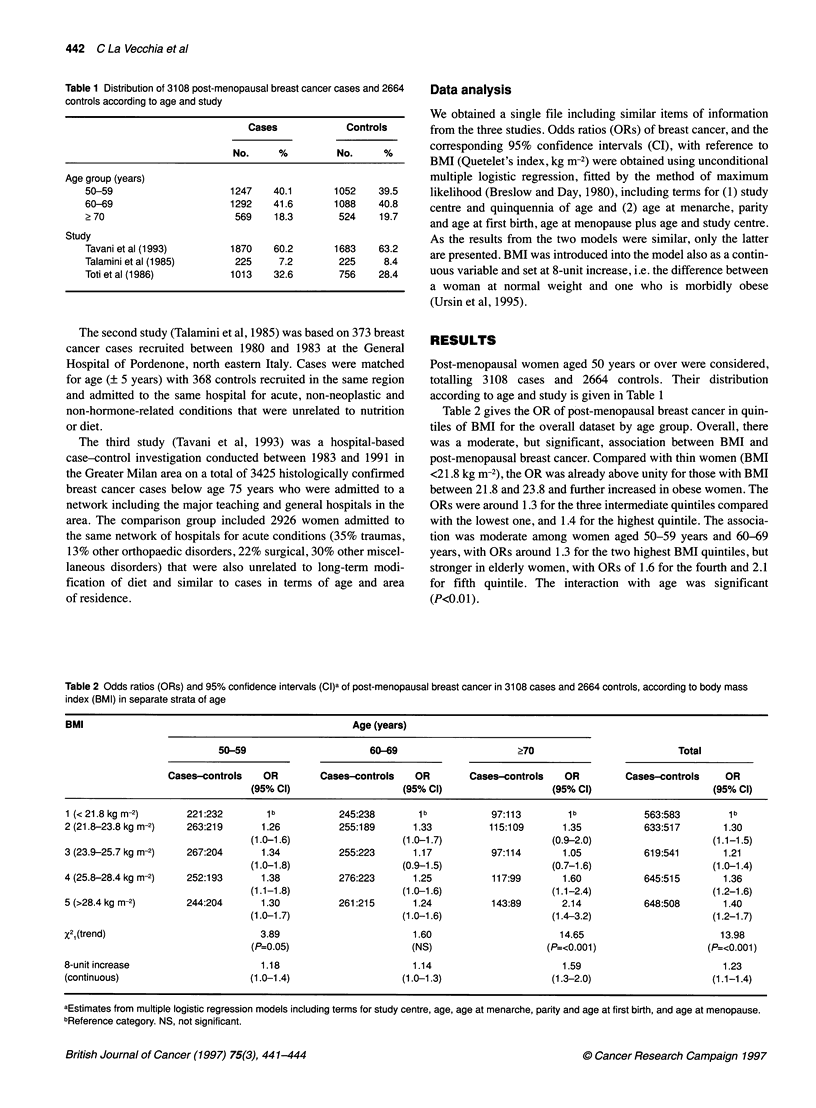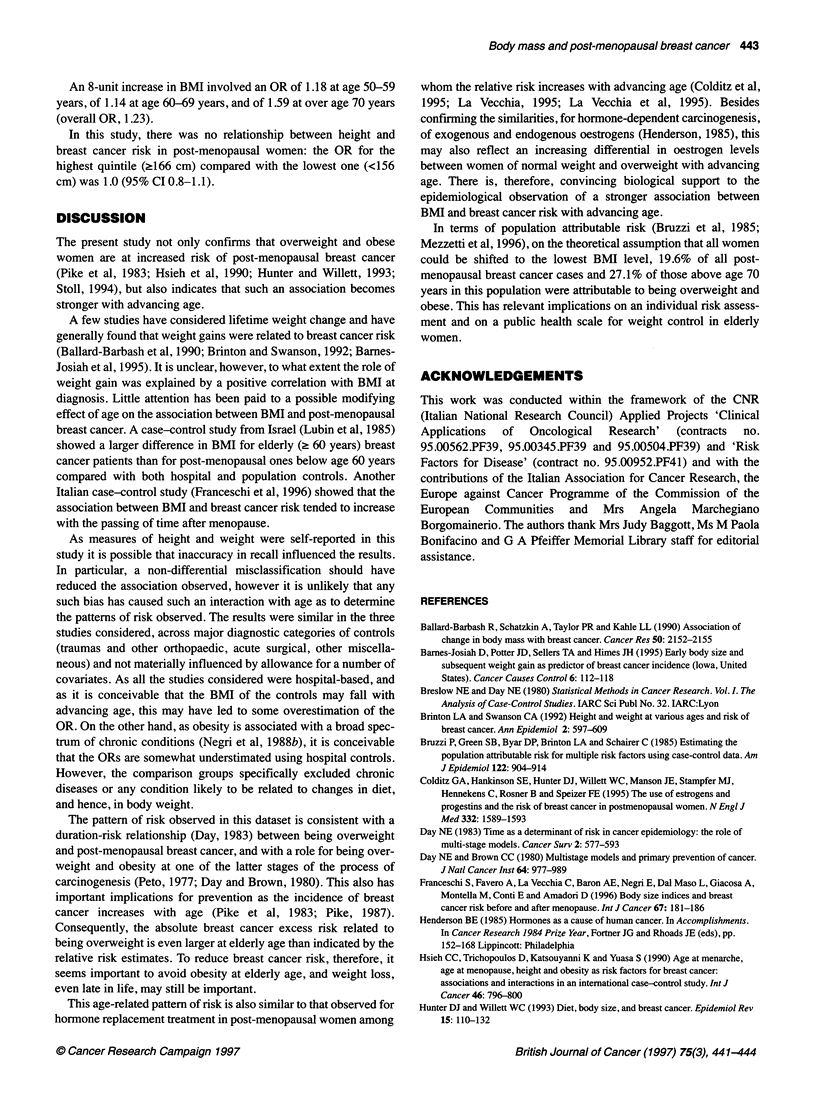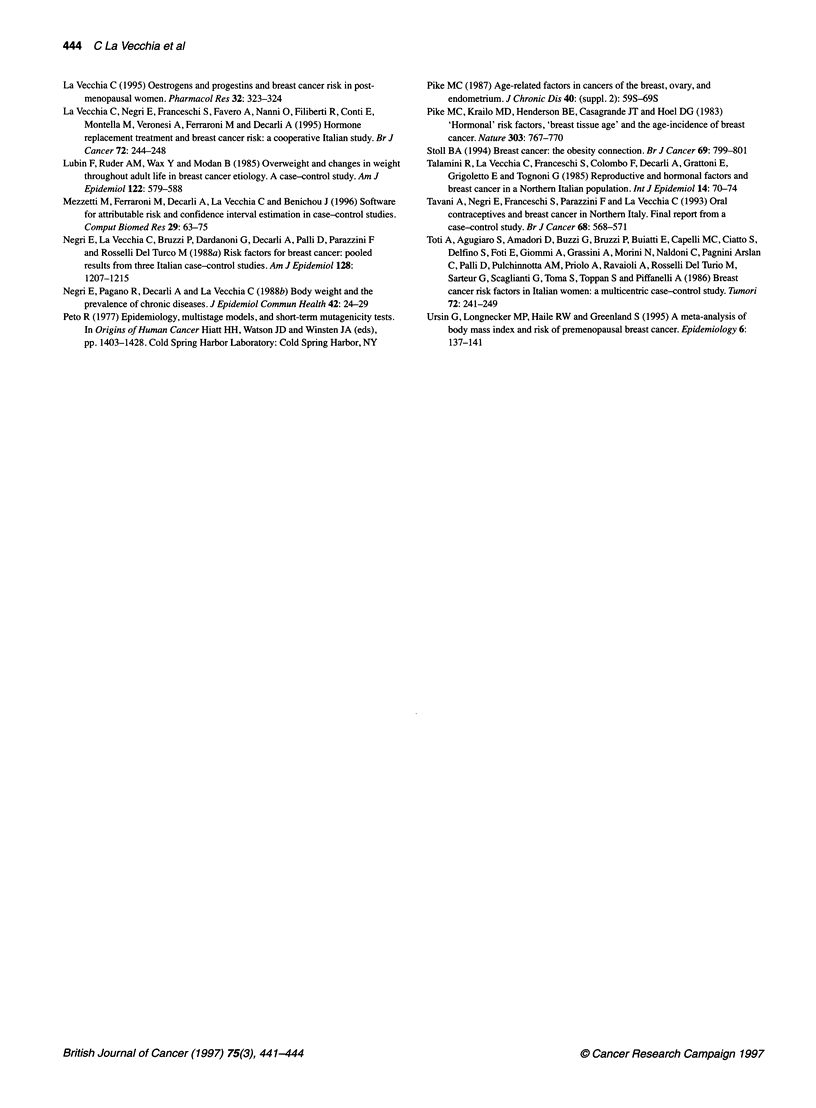# Body mass index and post-menopausal breast cancer: an age-specific analysis.

**DOI:** 10.1038/bjc.1997.73

**Published:** 1997

**Authors:** C. La Vecchia, E. Negri, S. Franceschi, R. Talamini, P. Bruzzi, D. Palli, A. Decarli

**Affiliations:** Istituto di Ricerche Farmacologiche Mario Negri, Milan, Italy.

## Abstract

The relationship between body mass index (BMI, Quetelet's index, kg m(-2)) and post-menopausal breast cancer risk was considered in age-specific strata on the basis of a pooled analysis of three Italian case-control studies, including a total of 3108 post-menopausal breast cancer patients aged 50 years or over and 2664 control subjects. Overall, there was a moderate, but significant, association between BMI and post-menopausal breast cancer: the odds ratios (ORs) were around 1.3 for the three intermediate quintiles compared with the lowest one, and 1.4 for the highest one. The association was moderate among women aged 50-59 years and 60-69 years, with ORs around 1.3 for the highest BMI quintiles, but stronger among elderly women, with ORs of 1.6 for the fourth and 2.1 for the fifth quintile. An 8-unit increase in BMI involved an OR of 1.18 at age 50-59 years, of 1.14 at age 60-69 years and of 1.59 above age 70 years. This pattern of risk is similar to that observed for post-menopausal hormone replacement treatment and is consistent with a duration-risk relationship in the exposure to high oestrogen levels and with a greater differential in oestrogen levels in overweight elderly women. In terms of population attributable risk, 19.6% of all post-menopausal breast cancer patients and 27.1% of those in women above age 70 years were attributable to overweight and obesity in this population. This has, therefore, major preventive implications as to reduce breast cancer risk late in life, it is essentially important to control weight gain in elderly women.


British Journal of Cancer (1997) 75(3), 441–444
© 1997 Cancer Research Campaign

### Body mass index and post-menopausal breast cancer: an age-specific analysis

C La Vecchia[1,2], E Negri[1], S Franceschi[3], R Talamini[3], P Bruzzi[4], D Palli[5], A Decarli[2]

[1]Istituto di Ricerche Farmacologiche 'Mario Negri', Via Eritrea 62, 20157 Milan, Italy; [2]Istituto di Biometria e Statistica Medica, Università degli Studi di Milano, Via Venezian 1, 20133 Milan, Italy; [3]Centro di Riferimento Oncologico, Via Pedemontana Occ. le, 33081 Aviano, Pordenone, Italy; [4]Istituto Nazionale per la Ricerca sul Cancro, Viale Benedetto XV 10, 16132 Genoa, Italy; [5]Centro per lo Studio e la Prevenzione Oncologica (CSPO), Viale A. Volta 171, 50131 Florence, Italy

**Summary** The relationship between body mass index (BMI, Quetelet's index, kg $m^{-2}$) and post-menopausal breast cancer risk was considered in age-specific strata on the basis of a pooled analysis of three Italian case–control studies, including a total of 3108 post-menopausal breast cancer patients aged 50 years or over and 2664 control subjects. Overall, there was a moderate, but significant, association between BMI and post-menopausal breast cancer: the odds ratios (ORs) were around 1.3 for the three intermediate quintiles compared with the lowest one, and 1.4 for the highest one. The association was moderate among women aged 50–59 years and 60–69 years, with ORs around 1.3 for the highest BMI quintiles, but stronger among elderly women, with ORs of 1.6 for the fourth and 2.1 for the fifth quintile. An 8-unit increase in BMI involved an OR of 1.18 at age 50–59 years, of 1.14 at age 60–69 years and of 1.59 above age 70 years. This pattern of risk is similar to that observed for post-menopausal hormone replacement treatment and is consistent with a duration–risk relationship in the exposure to high oestrogen levels and with a greater differential in oestrogen levels in overweight elderly women. In terms of population attributable risk, 19.6% of all post-menopausal breast cancer patients and 27.1% of those in women above age 70 years were attributable to overweight and obesity in this population. This has, therefore, major preventive implications as to reduce breast cancer risk late in life, it is essentially important to control weight gain in elderly women.

**Keywords:** body mass; breast neoplasm; epidemiology; risk

Being overweight and obese have been consistently associated with increased breast cancer risk in post-menopausal women. This is attributed to elevated levels or availability of circulating oestrogens in obese post-menopausal women due to the conversion of androgens to oestrogens in peripheral adipose tissue and to lower levels of sex hormone-binding globulin (SHBG) in overweight women (Pike et al, 1983; Hunter and Willett, 1993; Stoll, 1994).

A direct trend in risk has been observed between post-menopausal breast cancer and measures of body mass index (BMI) (Pike et al, 1983; Hsieh et al, 1990; Hunter and Willett, 1993; Stoll, 1994), but little attention has been paid to measures of the duration-risk relationship or to any other time factor. It is conceivable, in fact, that the association increases with prolonged exposure to overweight-related high oestrogen levels, i.e. with longer intervals after menopause. Furthermore, several studies on exogenous oestrogens (hormonal replacement treatment, HRT) and breast cancer have tended to show stronger associations with advancing age (Colditz et al, 1995; La Vecchia et al, 1995) For example the American Nurses' Health Study (Colditz et al, 1995) found a relative risk of 1.4 for long term (≥ 5 years) current users of HRT below age 55 years, but of 1.7 at age 60–64 years. This may also reflect a gradual increase in the difference of oestrogen levels between HRT users and non-users, as well as between women of normal weight and overweight women, with advancing age.

In a cooperative case–control study of 2566 cases and 2588 controls conducted in various Italian regions (Franceschi et al, 1996), the relative risk for the highest *vs* the lowest BMI quintile was 1.1 at age 50–59 years, 1.5 at age 60–69 years and 2.9 at age 70 years and over. This would suggest that the strength of the association between BMI and breast cancer risk increases with advancing age.

Given the relevance of this issue for individual risk assessment and to the public health, we considered the age-specific pattern of the relationship between BMI and breast cancer risk in post-menopausal women, using pooled data from three other Italian case–control studies.

*Received 3 July 1996*
*Revised 5 August 1996*
*Accepted 5 August 1996*

*Correspondence to:* C La Vecchia, Istituto di Ricerche Farmacologiche 'Mario Negri', Via Eritrea 62, 20157 Milan, Italy

### MATERIALS AND METHODS

The design and methods of the original investigations and their pooled analysis have been described in detail (Negri et al, 1988*a*). Briefly, the first study (Toti et al, 1986) was a cooperative hospital-based case–control investigation conducted between 1980 and 1983 in 13 breast cancer clinics from ten Italian provinces (eight from northern Italy, one from central and one from southern Italy). A total of 1556 incident, histologically confirmed cases were interviewed, together with 1473 controls admitted for acute, orthopaedic, medical or surgical conditions unrelated to risk factors for breast cancer and to long-term modification of diet (such as peptic ulcer and other gastric disorders, Crohn's disease or ulcerative colitis, diabetes mellitus, hyperuricaemia or any other metabolic condition).

**Table 1** Distribution of 3108 post-menopausal breast cancer cases and 2664 controls according to age and study

| | Cases | | Controls | |
|---|---|---|---|---|
| | No. | % | No. | % |
| Age group (years) | | | | |
| 50–59 | 1247 | 40.1 | 1052 | 39.5 |
| 60–69 | 1292 | 41.6 | 1088 | 40.8 |
| ≥ 70 | 569 | 18.3 | 524 | 19.7 |
| Study | | | | |
| Tavani et al (1993) | 1870 | 60.2 | 1683 | 63.2 |
| Talamini et al (1985) | 225 | 7.2 | 225 | 8.4 |
| Toti et al (1986) | 1013 | 32.6 | 756 | 28.4 |

The second study (Talamini et al, 1985) was based on 373 breast cancer cases recruited between 1980 and 1983 at the General Hospital of Pordenone, north eastern Italy. Cases were matched for age (± 5 years) with 368 controls recruited in the same region and admitted to the same hospital for acute, non-neoplastic and non-hormone-related conditions that were unrelated to nutrition or diet.

The third study (Tavani et al, 1993) was a hospital-based case–control investigation conducted between 1983 and 1991 in the Greater Milan area on a total of 3425 histologically confirmed breast cancer cases below age 75 years who were admitted to a network including the major teaching and general hospitals in the area. The comparison group included 2926 women admitted to the same network of hospitals for acute conditions (35% traumas, 13% other orthopaedic disorders, 22% surgical, 30% other miscellaneous disorders) that were also unrelated to long-term modification of diet and similar to cases in terms of age and area of residence.

### Data analysis

We obtained a single file including similar items of information from the three studies. Odds ratios (ORs) of breast cancer, and the corresponding 95% confidence intervals (CI), with reference to BMI (Quetelet's index, kg $m^{-2}$) were obtained using unconditional multiple logistic regression, fitted by the method of maximum likelihood (Breslow and Day, 1980), including terms for (1) study centre and quinquennia of age and (2) age at menarche, parity and age at first birth, age at menopause plus age and study centre. As the results from the two models were similar, only the latter are presented. BMI was introduced into the model also as a continuous variable and set at 8-unit increase, i.e. the difference between a woman at normal weight and one who is morbidly obese (Ursin et al, 1995).

### RESULTS

Post-menopausal women aged 50 years or over were considered, totalling 3108 cases and 2664 controls. Their distribution according to age and study is given in Table 1

Table 2 gives the OR of post-menopausal breast cancer in quintiles of BMI for the overall dataset by age group. Overall, there was a moderate, but significant, association between BMI and post-menopausal breast cancer. Compared with thin women (BMI <21.8 kg $m^{-2}$), the OR was already above unity for those with BMI between 21.8 and 23.8 and further increased in obese women. The ORs were around 1.3 for the three intermediate quintiles compared with the lowest one, and 1.4 for the highest quintile. The association was moderate among women aged 50–59 years and 60–69 years, with ORs around 1.3 for the two highest BMI quintiles, but stronger in elderly women, with ORs of 1.6 for the fourth and 2.1 for fifth quintile. The interaction with age was significant ($P$<0.01).

**Table 2** Odds ratios (ORs) and 95% confidence intervals (CI)[a] of post-menopausal breast cancer in 3108 cases and 2664 controls, according to body mass index (BMI) in separate strata of age

| BMI | Age (years) 50–59 | | 60–69 | | ≥70 | | Total | |
|---|---|---|---|---|---|---|---|---|
| | Cases–controls | OR (95% CI) | Cases–controls | OR (95% CI) | Cases–controls | OR (95% CI) | Cases–controls | OR (95% CI) |
| 1 (< 21.8 kg $m^{-2}$) | 221:232 | 1[b] | 245:238 | 1[b] | 97:113 | 1[b] | 563:583 | 1[b] |
| 2 (21.8–23.8 kg $m^{-2}$) | 263:219 | 1.26 (1.0–1.6) | 255:189 | 1.33 (1.0–1.7) | 115:109 | 1.35 (0.9–2.0) | 633:517 | 1.30 (1.1–1.5) |
| 3 (23.9–25.7 kg $m^{-2}$) | 267:204 | 1.34 (1.0–1.8) | 255:223 | 1.17 (0.9–1.5) | 97:114 | 1.05 (0.7–1.6) | 619:541 | 1.21 (1.0–1.4) |
| 4 (25.8–28.4 kg $m^{-2}$) | 252:193 | 1.38 (1.1–1.8) | 276:223 | 1.25 (1.0–1.6) | 117:99 | 1.60 (1.1–2.4) | 645:515 | 1.36 (1.2–1.6) |
| 5 (>28.4 kg $m^{-2}$) | 244:204 | 1.30 (1.0–1.7) | 261:215 | 1.24 (1.0–1.6) | 143:89 | 2.14 (1.4–3.2) | 648:508 | 1.40 (1.2–1.7) |
| $\chi^2_1$(trend) | | 3.89 ($P$=0.05) | | 1.60 (NS) | | 14.65 ($P$=<0.001) | | 13.98 ($P$=<0.001) |
| 8-unit increase (continuous) | | 1.18 (1.0–1.4) | | 1.14 (1.0–1.3) | | 1.59 (1.3–2.0) | | 1.23 (1.1–1.4) |

[a]Estimates from multiple logistic regression models including terms for study centre, age, age at menarche, parity and age at first birth, and age at menopause. [b]Reference category. NS, not significant.

© Cancer Research Campaign 1997

An 8-unit increase in BMI involved an OR of 1.18 at age 50–59 years, of 1.14 at age 60–69 years, and of 1.59 at over age 70 years (overall OR, 1.23).

In this study, there was no relationship between height and breast cancer risk in post-menopausal women: the OR for the highest quintile (≥166 cm) compared with the lowest one (<156 cm) was 1.0 (95% CI 0.8–1.1).

### DISCUSSION

The present study not only confirms that overweight and obese women are at increased risk of post-menopausal breast cancer (Pike et al, 1983; Hsieh et al, 1990; Hunter and Willett, 1993; Stoll, 1994), but also indicates that such an association becomes stronger with advancing age.

A few studies have considered lifetime weight change and have generally found that weight gains were related to breast cancer risk (Ballard-Barbash et al, 1990; Brinton and Swanson, 1992; Barnes-Josiah et al, 1995). It is unclear, however, to what extent the role of weight gain was explained by a positive correlation with BMI at diagnosis. Little attention has been paid to a possible modifying effect of age on the association between BMI and post-menopausal breast cancer. A case–control study from Israel (Lubin et al, 1985) showed a larger difference in BMI for elderly (≥ 60 years) breast cancer patients than for post-menopausal ones below age 60 years compared with both hospital and population controls. Another Italian case–control study (Franceschi et al, 1996) showed that the association between BMI and breast cancer risk tended to increase with the passing of time after menopause.

As measures of height and weight were self-reported in this study it is possible that inaccuracy in recall influenced the results. In particular, a non-differential misclassification should have reduced the association observed, however it is unlikely that any such bias has caused such an interaction with age as to determine the patterns of risk observed. The results were similar in the three studies considered, across major diagnostic categories of controls (traumas and other orthopaedic, acute surgical, other miscellaneous) and not materially influenced by allowance for a number of covariates. As all the studies considered were hospital-based, and as it is conceivable that the BMI of the controls may fall with advancing age, this may have led to some overestimation of the OR. On the other hand, as obesity is associated with a broad spectrum of chronic conditions (Negri et al, 1988*b*), it is conceivable that the ORs are somewhat understimated using hospital controls. However, the comparison groups specifically excluded chronic diseases or any condition likely to be related to changes in diet, and hence, in body weight.

The pattern of risk observed in this dataset is consistent with a duration-risk relationship (Day, 1983) between being overweight and post-menopausal breast cancer, and with a role for being overweight and obesity at one of the latter stages of the process of carcinogenesis (Peto, 1977; Day and Brown, 1980). This also has important implications for prevention as the incidence of breast cancer increases with age (Pike et al, 1983; Pike, 1987). Consequently, the absolute breast cancer excess risk related to being overweight is even larger at elderly age than indicated by the relative risk estimates. To reduce breast cancer risk, therefore, it seems important to avoid obesity at elderly age, and weight loss, even late in life, may still be important.

This age-related pattern of risk is also similar to that observed for hormone replacement treatment in post-menopausal women among whom the relative risk increases with advancing age (Colditz et al, 1995; La Vecchia, 1995; La Vecchia et al, 1995). Besides confirming the similarities, for hormone-dependent carcinogenesis, of exogenous and endogenous oestrogens (Henderson, 1985), this may also reflect an increasing differential in oestrogen levels between women of normal weight and overweight with advancing age. There is, therefore, convincing biological support to the epidemiological observation of a stronger association between BMI and breast cancer risk with advancing age.

In terms of population attributable risk (Bruzzi et al, 1985; Mezzetti et al, 1996), on the theoretical assumption that all women could be shifted to the lowest BMI level, 19.6% of all post-menopausal breast cancer cases and 27.1% of those above age 70 years in this population were attributable to being overweight and obese. This has relevant implications on an individual risk assessment and on a public health scale for weight control in elderly women.

### ACKNOWLEDGEMENTS

This work was conducted within the framework of the CNR (Italian National Research Council) Applied Projects 'Clinical Applications of Oncological Research' (contracts no. 95.00562.PF39, 95.00345.PF39 and 95.00504.PF39) and 'Risk Factors for Disease' (contract no. 95.00952.PF41) and with the contributions of the Italian Association for Cancer Research, the Europe against Cancer Programme of the Commission of the European Communities and Mrs Angela Marchegiano Borgomainerio. The authors thank Mrs Judy Baggott, Ms M Paola Bonifacino and G A Pfeiffer Memorial Library staff for editorial assistance.

### REFERENCES

Ballard-Barbash R, Schatzkin A, Taylor PR and Kahle LL (1990) Association of change in body mass with breast cancer. *Cancer Res* **50**: 2152–2155

Barnes-Josiah D, Potter JD, Sellers TA and Himes JH (1995) Early body size and subsequent weight gain as predictor of breast cancer incidence (Iowa, United States). *Cancer Causes Control* **6**: 112–118

Breslow NE and Day NE (1980) *Statistical Methods in Cancer Research. Vol. I. The Analysis of Case-Control Studies*. IARC Sci Publ No. 32. IARC:Lyon

Brinton LA and Swanson CA (1992) Height and weight at various ages and risk of breast cancer. *Ann Epidemiol* **2**: 597–609

Bruzzi P, Green SB, Byar DP, Brinton LA and Schairer C (1985) Estimating the population attributable risk for multiple risk factors using case-control data. *Am J Epidemiol* **122**: 904–914

Colditz GA, Hankinson SE, Hunter DJ, Willett WC, Manson JE, Stampfer MJ, Hennekens C, Rosner B and Speizer FE (1995) The use of estrogens and progestins and the risk of breast cancer in postmenopausal women. *N Engl J Med* **332**: 1589–1593

Day NE (1983) Time as a determinant of risk in cancer epidemiology: the role of multi-stage models. *Cancer Surv* **2**: 577–593

Day NE and Brown CC (1980) Multistage models and primary prevention of cancer. *J Natl Cancer Inst* **64**: 977–989

Franceschi S, Favero A, La Vecchia C, Baron AE, Negri E, Dal Maso L, Giacosa A, Montella M, Conti E and Amadori D (1996) Body size indices and breast cancer risk before and after menopause. *Int J Cancer* **67**: 181–186

Henderson BE (1985) Hormones as a cause of human cancer. In *Accomplishments. In Cancer Research 1984 Prize Year*, Fortner JG and Rhoads JE (eds), pp. 152–168 Lippincott: Philadelphia

Hsieh CC, Trichopoulos D, Katsouyanni K and Yuasa S (1990) Age at menarche, age at menopause, height and obesity as risk factors for breast cancer: associations and interactions in an international case–control study. *Int J Cancer* **46**: 796–800

Hunter DJ and Willett WC (1993) Diet, body size, and breast cancer. *Epidemiol Rev* **15**: 110–132

© Cancer Research Campaign 1997

La Vecchia C (1995) Oestrogens and progestins and breast cancer risk in post-menopausal women. *Pharmacol Res* **32**: 323–324

La Vecchia C, Negri E, Franceschi S, Favero A, Nanni O, Filiberti R, Conti E, Montella M, Veronesi A, Ferraroni M and Decarli A (1995) Hormone replacement treatment and breast cancer risk: a cooperative Italian study. *Br J Cancer* **72**: 244–248

Lubin F, Ruder AM, Wax Y and Modan B (1985) Overweight and changes in weight throughout adult life in breast cancer etiology. A case–control study. *Am J Epidemiol* **122**: 579–588

Mezzetti M, Ferraroni M, Decarli A, La Vecchia C and Benichou J (1996) Software for attributable risk and confidence interval estimation in case–control studies. *Comput Biomed Res* **29**: 63–75

Negri E, La Vecchia C, Bruzzi P, Dardanoni G, Decarli A, Palli D, Parazzini F and Rosselli Del Turco M (1988*a*) Risk factors for breast cancer: pooled results from three Italian case–control studies. *Am J Epidemiol* **128**: 1207–1215

Negri E, Pagano R, Decarli A and La Vecchia C (1988*b*) Body weight and the prevalence of chronic diseases. *J Epidemiol Commun Health* **42**: 24–29

Peto R (1977) Epidemiology, multistage models, and short-term mutagenicity tests. In *Origins of Human Cancer* Hiatt HH, Watson JD and Winsten JA (eds), pp. 1403–1428. Cold Spring Harbor Laboratory: Cold Spring Harbor, NY

Pike MC (1987) Age-related factors in cancers of the breast, ovary, and endometrium. *J Chronic Dis* **40**: (suppl. 2): 59S–69S

Pike MC, Krailo MD, Henderson BE, Casagrande JT and Hoel DG (1983) 'Hormonal' risk factors, 'breast tissue age' and the age-incidence of breast cancer. *Nature* **303**: 767–770

Stoll BA (1994) Breast cancer: the obesity connection. *Br J Cancer* **69**: 799–801

Talamini R, La Vecchia C, Franceschi S, Colombo F, Decarli A, Grattoni E, Grigoletto E and Tognoni G (1985) Reproductive and hormonal factors and breast cancer in a Northern Italian population. *Int J Epidemiol* **14**: 70–74

Tavani A, Negri E, Franceschi S, Parazzini F and La Vecchia C (1993) Oral contraceptives and breast cancer in Northern Italy. Final report from a case–control study. *Br J Cancer* **68**: 568–571

Toti A, Agugiaro S, Amadori D, Buzzi G, Bruzzi P, Buiatti E, Capelli MC, Ciatto S, Delfino S, Foti E, Giommi A, Grassini A, Morini N, Naldoni C, Pagnini Arslan C, Palli D, Pulchinnotta AM, Priolo A, Ravaioli A, Rosselli Del Turio M, Sarteur G, Scaglianti G, Toma S, Toppan S and Piffanelli A (1986) Breast cancer risk factors in Italian women: a multicentric case–control study. *Tumori* **72**: 241–249

Ursin G, Longnecker MP, Haile RW and Greenland S (1995) A meta-analysis of body mass index and risk of premenopausal breast cancer. *Epidemiology* **6**: 137–141

 © Cancer Research Campaign 1997